# Factors Associated with Activities of Daily Life Disability among Centenarians in Rural Chongqing, China: A Cross-Sectional Study

**DOI:** 10.3390/ijerph14111364

**Published:** 2017-11-09

**Authors:** Tingting Wu, Lu Lu, Li Luo, Yingqi Guo, Liying Ying, Qingliu Tao, Huan Zeng, Lingli Han, Zumin Shi, Yong Zhao

**Affiliations:** 1 School of Public Health and Management, Chongqing Medical University, Chongqing 400016, China; m17749945967@163.com (T.W.); kkllu001@126.com (L.Lu); sky501230032@sina.com (L.Luo); zenghuan586@aliyun.com (H.Z.); 13452410655@163.com (L.H.); 2 Research Center for Medicine and Social Development, Chongqing Medical University, Chongqing 400016, China; 3 Collaborative Innovation Center of Social Risks Governance in Health, Chongqing Medical University, Chongqing 400016, China; 4 Department of Social Work and Social Administration, the University of Hong Kong, Hong Kong, China; guoyingqi.hk@hotmail.com; 5 School of Nursing, Zhejiang Chinese Medical University, Hangzhou 310000, China; ly195024@163.com; 6 Chongqing Health Education Institute, Chongqing 404000, China; taoqingliu2017@163.com; 7 Adelaide Medical School, University of Adelaide, Adelaide 5000, Australia; zumin.shi@adelaide.edu.au

**Keywords:** ADL disability, centenarians, lifestyle, rural Chongqing

## Abstract

Objective: This study aims to ascertain the health and lifestyle factors associated with the activities of daily living (ADL) disability of centenarians in rural Chongqing, China. Method: 564 centenarians living in rural Chongqing were selected for this cross-sectional study. Demographic characteristics and self-reported lifestyle factors were obtained from face-to-face interviews. ADL disability was measured using the Katz Activities of Daily Living Scale. Result: Among the respondents, 65.7% were considered ADL disability centenarians. Multivariable logistic regression analysis showed that preference for salt, drinking habits, social activities, physical activity, and failure to follow good diet habits were significantly associated with the ADL disability of centenarians. Conclusion: ADL disability of centenarians was associated with certain lifestyle habits. This outcome suggested that target intervention may help maintain ADL independence even among the oldest of the elderly population.

## 1. Introduction

As aging intensifies, it causes numerous social problems, including those related to health care and shelter for the elderly, that situation is also very serious in Chongqing, China. Chongqing, located in Southwest China, is a fast-growing city with a total area of 824 thousand square kilometres and a permanent population of 3371.84 million [1]. Its population density varies substantially, (i.e., 2.8 thousand person/km^2^ in the Yuzhong district and 57 person/km^2^ in the Chenkou county). A clear disparity exists between its urban and rural areas. Data from the Chongqing Municipal Bureau show that Chongqing became an aging society in 2000, and the proportions of people aged 65 and over from 2013 to 2015 are 11.88%, 12.02%, and 12.17%, respectively [1]. To alleviate related problems, it is necessary to study the aging population.

Centenarians should be the focus of attention, as their morbidity is higher and their self-care ability is lower relative to the average elderly. Therefore, they are strongly dependent on society and others [2]. Having a centenarian relative is not unusual in the 21st century. The United Nations reported that the total centenarians in the world in 2009 was approximately 455,000, and was estimated to be 3.4 million by 2050 [3]. The Gerontological Society of China stated that the country had over 47,000 centenarians in 2012 [4]. The life expectancy of people in many countries is also increasing. As a symbol of extreme longevity, many previous studies have focused on centenarians to identify determinants related to super-longevity from variable factors such as biochemistry, genetics, nutrition, and lifestyles [5,6,7,8].

Activities of daily living (ADLs) are the essential behaviors that prove the capability of a person to live independently [9]. Understanding the status of ADLs in extremely elderly populations is gaining importance [10]. Prior research revealed that the status of ADLs or functionality among centenarians was poor [11,12,13]. These findings may indicate that the increasing number of centenarians is associated with a large population of the extremely elderly suffering from disability, eventually leading to higher health care demand and costs [14]. ADL disability places a high burden on individuals, care professionals, and health care systems [15]. Especially in China, younger family members are obliged to care for their older relatives, and the offspring may bear onerous responsibilities to support ADL-disabled centenarians [16,17]. Effective interventions that prevent disability can reduce such burdens. The development of such interventions and the identification of people who might benefit from them require the determination of factors that predict ADL disability [18].

Extant literature identified several factors of physical function and disability in elders [19,20,21,22,23,24]. For centenarians, some determinants were also associated with ADLs or overall status. A few studies have recently ascertained that lifestyle habits, such as physical activity, social activity, eating protein-rich food, and living with family members were associated with ADLs or functional disability, whereas smoking and alcohol consumption were not [12,13,14]. However, most of these investigations were conducted in developed countries. Few examinations focus on the factors associated with ADL disability among large sample centenarians in China, especially in rural areas. A clear economic disparity exists between urban and rural areas. This study aims to identify associations between lifestyle factors and ADL disability among centenarians in rural Chongqing, China, and provide reference for the government to formulate the measures related to the management and service of centenarians.

## 2. Materials and Methods

### 2.1. Study Population and Method

The design and methods of this research (including the survey administration, pretest, and methods of data collection) have been reported previously [7]. This work was recommended by the Chongqing Health Education Institute, the Local Health Education Institute and the Centre for Disease Control and Prevention (CDC). The census of centenarians was conducted in nine districts of Chongqing City (29.19 million population) and 41 affiliated surrounding counties from 1 September to 31 December in 2010. Sampling was performed by screening and reviewing the identification cards of participants.

A total of 877 individuals (173 men and 704 women) were identified as aged 100 or above. Examining the hukou and current residences of all participants led to the choice of 606 centenarians who had rural hukou as the target sample. Hukou is the household registration system in China [25]. People were categorized as urban or rural residents according to their hukou. When enforced strictly, hukou could define one’s living residence and occupation [25,26]. In this survey, the current residences of the centenarians were reviewed by investigators from local CDCs familiar with the distribution of urban and rural areas. On the basis of such review, most of the centenarians were ascertained to have maintained the residences recorded in their hukou. 

**Figure 1:**
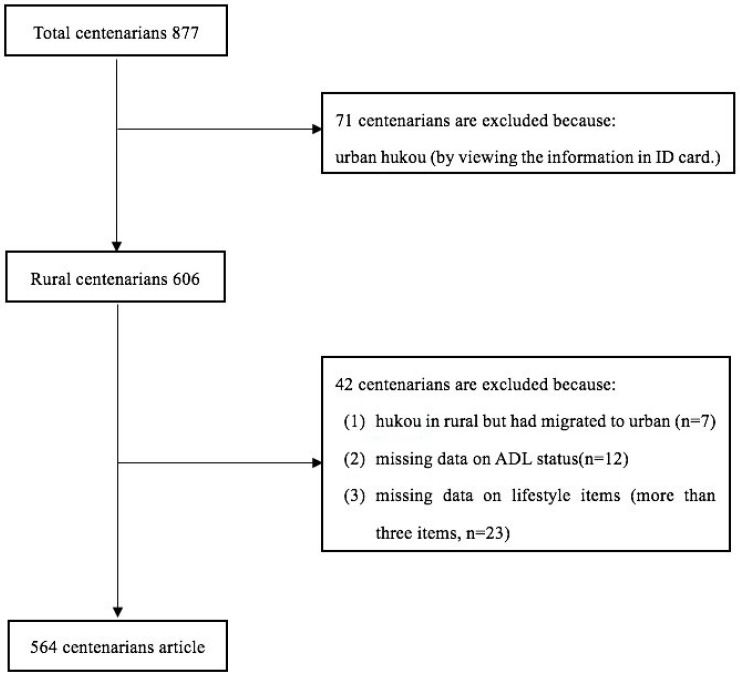
Flowchart of the stepwise selection process of centenarians.

Exclusion criteria of this study include the following: (1) centenarians with rural hukou but who migrated to urban areas, (2) participants with missing data on ADL status, and (3) respondents with missing data on lifestyle items (more than three items). Forty-two participants were excluded, including seven centenarians who were not considered “real” rural centenarians, 12 individuals with missing ADL status, and 23 individuals with more than three lifestyle items whose data were missing. Finally, 564 centenarians (93 males and 471 females) were included in our survey (see Figure 1 for details).

### 2.2. Ethics Approval

This research was performed in accordance with the Declaration of Helsinki. In addition, this study was approved by the Ethical Committee of Chongqing Medical University (ethical approval code: 2015008). All participants were informed of the purpose of the study and their cooperation was voluntary. All participants were interviewed and provided oral consent. Consent was also obtained for the examination of the participants’ ID cards and hukou. To ensure anonymity, their names were not required in the questionnaire. Instead, each centenarian was given a unique code that was written on the questionnaire. An ethical clearance was also obtained from the local health authorities in Chongqing.

### 2.3. Measures

Face-to-face interview questionnaires were administered by well-trained researchers from local health education institutes and CDCs to collect information on demographic characteristics, health conditions, self-reported lifestyle behaviors, and ADL status. In rare cases (e.g., participants with severe cognitive impairment), relatives of the participants were interviewed.

#### 2.3.1. Demographic Characteristics and Health Condition

Demographic characteristics included age, gender, and ethnicity. Date of birth and ethnicity were ascertained from national ID cards. Ethnicity was categorized as Han or ethnic minority. 

Participants were asked whether they had any current chronic conditions including cancer, stroke, heart diseases, dementia, diabetes, hypertension, osteoarthritis, cardiovascular disease, respiratory disease, and vision problems.

#### 2.3.2. Self-Reported Lifestyle Behaviors

Self-reported lifestyle behaviors included living arrangement, smoking, drinking habit, regular diet, avoiding sweet, high-cholesterol, and fatty food, salt preference, social activities, physical activity, reading, and watching TV or listening to radio. Living arrangement was classified as living alone and living with others. Participants were identified as smokers or non-smokers. Note that both current smokers and ex-smokers were defined as smokers. Drinking was classified into “no drinking” and “regular” drinking. The latter denoted alcohol consumption of more than three times per week for at least one year. Good diet habits refer to having various types of food including staple food, vegetable and/or fruits, and protein-rich food. Information on the diet habits of participants was determined through the following three questions: (1) “Do you eat staple food in every meal?”; (2) “Do you eat a vegetable and/or fruit every day?”; and (3) “Do you eat protein-rich food such as meat, fish, egg, dairy and soybean every day?” Those who reported in the affirmative for all items were categorized as having good diet habits. Salt preference was assessed through two questions: (1) “Do you like salty food such as pickles, preserved meat and salted fish?” and (2) “Do you add extra salt or salt seasoning while having meals?" Participants who responded in the affirmative for at least one of the two items were classified as having salt preference. Social activities were described as visiting or communicating with others, visiting community centers, and participating in recreational activities, such as playing cards or mah-jong (Chinese game played by four people with 144 tiles) with others. Frequency of social activities was classified into three levels: never or rarely (less than once per week), sometimes (1–2 times per week), and often (more than twice per week). In our study, we dichotomized responses for social participation into “yes” or “no” categories: “yes” if the answer was “sometimes” or “often” and “no” if the answer was “never or rarely.” Information on physical activity was collected through the question: “Do you often do housework or have regular physical exercise such as taking a walk, jogging, and Qigong (a system of breathing and exercise designed to benefit both physical and mental health)?” Yes or no answers were recorded accordingly.

#### 2.3.3. ADL Status

The ADL status of centenarians was obtained from interviews. The Katz Activities of Daily Living Scale [27] was utilized to evaluate their ADL. The ADL items include six self-care activities including eating, dressing, bathing, grooming, walking, and using the toilet. ADL disability was denoted as impairment in one or more of the six ADL items [28]. Centenarians constitute a special group, their language, cognition, and ability to understanding various aspects may differ from other age clusters. Quantifying their ADL using the same scale would be too difficult. In our study, participants were given three choices when they were asked about the six self-care ADL items: “can do,” “can do it with some difficulty,” and “cannot do it at all without help.” If any one answer of the six items is “cannot do it at all without help,” the participants were defined as impaired, and this question subject then ended. ADL items were dichotomized into two categories: “yes” if the answer was “can do” or “can do it with some difficulty,” and “no” if the answer was “cannot do it at all without help.”

### 2.4. Statistical Analyses

All survey data were encoded using Epidata 3.0. Statistical software (SAS version 9.0, SAS Institute, Cary, NC, USA) was utilized for statistical data analysis. A Pearson chi-square test was used to compare all demographic and lifestyle characteristics between the ADL-able group and ADL-disabled groups. Logistic regression analysis was conducted to assess the association between such characteristics and ADL disability. The factors associated with ADL disability were examined through bivariate and multivariable logistic regression models. Statistical significance was set at 0.05 (two sides). 

## 3. Results

### 3.1. Distribution of Characteristics

Table 1 shows the distribution of participant characteristics by absence or presence of ADL disability. In this study, 370 (65.6%) centenarians had ADL disability. Participants with ADL disability were more likely to be female (*p* = 0.031). The prevalence of ADL disability was significantly higher in the participants who reported not having good diet habits (*p* = 0.003), having a salt preference (*p* = 0.028), drinking (*p* = 0.000), not participating in social activities (*p* = 0.011), and lacking physical activities (*p* = 0.005) (Table 1). 

### 3.2. Bivariate Logistic Regression Analysis

Table 2 shows the results of the gender-adjusted bivariate logistic regression analysis, which indicated that four lifestyle factors remained significantly associated with better ADL status (Table 2). Participants who keep good diet habits (OR = 0.543, 95% CI [0.370–0.798]), participate in social activities (OR = 0.588, 95% CI [0.304–0.844], and participate in physical activities (OR = 0.561, 95% CI [0.373–0.845] were more likely to have better ADL status. Participants who had salt preference (OR = 3.016, 95%, CI [1.026–8.867]) were more likely to suffer ADL disability.

### 3.3. Multivariable Logistic Regression Analysis

The results of multivariable logistic regression analysis, which are similar to the results of bivariate analysis, indicated that females and participants who have current disease were more likely to suffer ADL disability, and four lifestyle factors remained significantly associated with better ADL status (Table 3). Participants who kept good diet habits (OR = 0.489, 95% CI [0.322–0.741]) were more likely to have better ADL status. Participants who had a salt preference (OR = 3.858, 95% CI [1.142–11.256]) were more likely to suffer ADL disability. Participants who participated in social activities (OR = 0.543, 95% CI [0.328–0.900] and physical activities (OR = 0.551, 95% CI [0.336–0.904]) were more likely to have better ADL status.

## 4. Discussion

The findings and their implications should be examined in the broadest context possible. Future research directions may also be highlighted. Results in the present study indicated an association between ADL disability and several lifestyle factors among centenarians in rural Chongqing. Females were more likely to be ADL-disabled. Chronic diseases increased the likelihood of ADL disability. The absence of good diet habits in centenarians was associated with ADL disability. Light drinking was strongly linked with ADL independence among the respondents. Participants who preferred salty food had poorer ADL functionality relative to those without such preference. Lack of socialization with others was also related to ADL dependence among rural centenarians. Centenarians who lacked physical activities also had poorer ADL ability than their active counterparts. 

Female centenarians in rural Chongqing were more likely to be ADL-disabled. This finding is consistent with previous studies [13,29]. A reason may be that physical function deteriorated faster in females [30]. Furthermore, only 93 (16.5%) centenarians in our study were males. Possibly, more males than females who suffered ADL disability have died before becoming centenarians.

Chronic disease was associated with ADL disability among the oldest of the study population. Previous studies have reported a close association between chronic condition and physical decline or disability in elders [31,32]. The current findings are in agreement with that of a Korean study that centenarians suffering from chronic diseases were at least 2.50 times more likely to suffer ADL disability [13]. These outcomes suggest the importance of medical care and self-management for chronic diseases and health conditions to maintain physical health among centenarians in rural Chongqing. 

Failing to maintain good diet habits was linked with ADL disability among centenarians. Food is beneficial for the physical performance of the elderly. Existing literature found that dairy products, vegetables, and fruits are beneficial for physical function in elders [33]. Eating more protein-rich food, such as meat and fish, is also good for the overall health of centenarians [12]. Moreover, adhering to a balanced diet and food diversity were essential for ADL independence. Elders who failed to maintain a balanced diet, such as the Mediterranean diet, or who lacked food diversity, were more likely to suffer ADL impairment or disability [34,35,36]. Hence, our findings suggested that following good diet habits may be crucial to avoiding ADL disability even in the extremely elderly population.

Centenarians with salt preference are more likely to be ADL disability. To our knowledge, few studies have investigated the association between salt preference and ADL disability in a long-lived population. Two possible explanations for the association are as follows. First, centenarians who preferred the salty taste may have excessive salt intake. Such high intake is a direct risk factor for many chronic diseases, including cardiovascular diseases and strokes, resulting in disability [16,37]. Second, high salt intake was linked to the decline of muscle mass and strength [38], which may result in poor physical function [39]. However, because of the lack of information on the body composition of participants, caution must be taken when interpreting the results in the present study. 

Lack of physical activity was associated with ADL disability among centenarians in rural Chongqing. It was known that physical activity benefits the physical health and functionality of elders. As a recent article reports, the elevated level of physical activity among Sardinian centenarians was associated with high ADL scores [40]. Our findings were in accordance with previous centenarian studies in Japan and South Korea, which found that centenarians who lacked physical activity had poor ADL capability [12,13]. However, identifying the causal relationship between physical activity and ADL disability from the current cross-sectional study is not possible. 

Lack of social activities was also linked to increasing odds of ADL disability. This finding is consistent with a previous centenarian study in Korea [13]. Extant literature indicated that social participation helps maintain physical health [41]. Having social support and engaging with others may help prevent illness and functional dependency in elders and centenarians [42,43]. Thus, socializing with others may be important for ADL performance of centenarians.

Any study of centenarians must take into account age inflation [44,45]. We consider the ages of centenarians by viewing their ID cards and asking their relatives. If a participant’s birth year were exaggerated in their ID cards, their stated age would not be correct. But this should be a very unlikely situation since it is important for Chinese people to report their accurate date of birth when making decisions on important life events such as match making for marriage, date of marriage, the date to start building a house, and other events [46]. Previous research has reported that age reporting is relatively good among Han Chinese, whereas age exaggeration is likely in ethnic minority age reporting [45,46,47]. Han Chinese, even if illiterate, can provide a reliable date of birth for themselves or their family members [46]. In our study, only 6.4% (36 participants) of the participants were ethnic minorities. This suggests that some of the participants who are ethnic minorities may not be centenarians. However, this is not a substantial bias. 

### Limitations

Our study has several limitations. First, this research employs a cross-sectional approach and cannot identify the causal relationship between lifestyle factors and ADL ability. Further longitudinal studies are necessary to confirm the relationship between lifestyle and ADL ability among centenarians. Second, in our study, impairment was defined as “cannot do it at all without help.” The ADL items were dichotomized into two categories, yes and no, and we did not record the specific options for these six themes. This lack of detail may have excluded some ADL disability, which means that these results might not recur in other age populations. In rare cases (e.g., participants with severe cognitive impairment), relatives of the participants were interviewed. We did not record this proportion, and such omission may cause information bias. Third, information of diet habits was based only on self-reported frequency intake of food items. The quantity of the food and nutrients was not analyzed. Furthermore, we asked about salt preference, but did not investigate their actual salt intake. Food preference might be an important determinant of food intake, but the two variables are different. Thus, further research is warranted to collect more detailed information on these two items in Chongqing’s centenarians. Fourth, due to design limitations, some confounding factors, such as education level, body mass index, and cognitive status, were not investigated. Previous studies have found that cognitive abilities are associated with physical disability [48]. Body mass index and waist circumference has also been associated with ADL disability [49]. Further studies should be considered in light of these factors. Finally, considering the diversity of customs and the imbalanced economic development in China, although there was a relatively large sample of 564 rural centenarians in our study, its generalizability may not accurately represent the entire population among all rural centenarians in China. 

## 5. Conclusions

In this research, centenarians in rural Chongqing, China, with beneficial lifestyle habits (including good diet habits, salt preference, no drinking, physical activity, and social activities) had better ADL status. This finding indicates that targeted intervention may help maintain ADL independence even among the oldest in the elderly population. Further longitudinal studies should be conducted to elucidate the factors associated with ADL independence in the extremely elderly population.

## Figures and Tables

**Table 1:** Distribution of characteristics among centenarians in rural Chongqing, China (*N* = 564).

Characteristics	No ADL Disability		ADL Disability		*p*-Value
	*n*	%	*n*	%	
**Demographic characteristics**					
Age					0.234
100–105	180	92.7	332	89.7	
106–110	14	7.3	38	10.3	
Gender					0.031 *
Male	41	21.1	52	14.1	
Female	153	78.9	318	85.9	
Ethnicity					0.595
Han	183	94.3	344	93.2	
Ethnic minority	11	5.7	26	6.8	
Current diseases					0.001 **
No	144	74.2	161	43.5	
Yes	50	25.8	209	56.5	
**Lifestyle factors**					
Living arrangement					0.061
Living alone	2	1.0	14	3.8	
Living with others	192	99.0	356	96.2	
Smoking					0.938
Yes	19	9.8	37	10.0	
No	175	90.2	333	90.0	
Drinking habits					0.000 *
No	161	83.0	307	83.0	
Yes	33	17.0	63	17.0	
Good diet habits					0.003 **
Yes	144	74.2	225	60.8	
No	50	25.8	145	39.2	
Avoiding sweet, fatty and high cholesterol food					0.22
Yes	17	8.8	45	12.2	
No	177	91.2	325	87.8	
Salt preference					0.028 *
Yes	4	0.5	23	6.2	
No	190	99.5	347	93.8	
Social activities					0.011 *
Yes	50	25.8	62	16.8	
No	144	74.2	308	83.2	
Physical activity					0.005 **
Yes	56	28.9	68	18.4	
No	138	71.1	301	82.6	
Reading					0.077
Yes	7	3.6	5	0.8	
No	187	96.4	363	99.2	
Watching TV or listening to radio					0.061
Yes	61	31.4	146	39.5	
No	133	68.6	224	60.5	

Note: (1) Abbreviations: ADL: activities of daily living. (2) * *p* < 0.05; ** *p* < 0.01.

**Table 2:** Bivariate logistic regression analysis for the association between factors and ADL disability.

Variables	OR	95% CI	*p*-Value
Age			
100–105	1		
106–110	1.366	0.718–2.600	0.342
Ethnicity			
Han nationality	1		
National minority	1.318	0.633–2.742	0.461
Present diseases			
No	1		
Yes	3.773	2.570–5.539	0.000 **
Living arrangement			
Living with others	1		
Living alone	3.861	0.865–17.236	0.077
Smoking			
Yes	1		
No	0.644	0.326–1.269	0.203
Drinking habits			
No	1		
Yes	1.171	0.719–1.906	0.526
Good diet habits			
Yes	0.543	0370–0.798	0.002 **
No	1		
Avoiding sweet, fatty and high cholesterol food			
Yes	1.429	0.793–2.576	0.235
No	1		
Salt preference			
No	1		
Yes	3.016	1.026–8.867	0.045 *
Social activities			
Yes	0.588	0.304–0.844	0.014 *
No	1		
Physical activity			
Yes	0.561	0.373–0.845	0.006 **
No	1		
Reading			
Yes	0.395	0.123–1.272	0.120
No	1		
Watching TV or listening to radio			
No			
Yes	1.413	0.977–2.044	0.066

Note: (1) Abbreviations: ADL: activities of daily living; OR: odds ratio; CI: confidence interval. (2) * *p* < 0.05; ** *p* < 0.01. (3) Bivariate logistic regression was adjusted for gender.

**Table 3:** Multivariable logistic regression analysis for the association between factors and ADL disability.

Variables	OR	95% CI	*p*-Value
Age			
100–105	1		
106–110	0.843	0.415–1.714	0.637
Gender			
Male	1		
Female	1.921	1.072–3.442	0.028 *
Ethnicity			
Han	1		
Ethnic minority	1.422	0.639–3.163	0.388
Present diseases			
No	1		
Yes	3.581	2.357–5.442	0.000 **
Living arrangement			
Living with others	1		
Living alone	3.259	0.653–16.277	0.150
Smoking			
Yes	1		
No	0.516	0.217–1.228	0.135
Drinking habits			
No	1		
Yes	0.746	0.398–1.397	0.360
Good diet habits			
Yes	0.489	0.322–0.741	0.001 **
No	1		
Avoiding sweet, fatty and high cholesterol food			
Yes	1.213	0.624–2.359	0.569
No	1		
Salt preference			
No	1		
Yes	3.585	1.142–11.256	0.029 *
Social activities			
Yes	0.543	0.328–0.900	0.018 *
No	1		
Physical activity			
Yes	0.551	0.336–0.904	0.018 *
No	1		
Reading			
Yes	0.645	0.181–2.293	0.498
No	1		
Watching TV or listening to radio			
No	1		
Yes	0.880	0.557–1.392	0.585

Note: (1) Abbreviations: ADL: activities of daily living; OR: odds ratio; CI: confidence interval. (2) * *p* < 0.05; ** *p* < 0.01. (3) Multivariate logistic regression was adjusted for age, gender, ethnicity, present diseases, living arrangement, smoking, drinking habits, good diet habits, avoiding sweet, fatty, and high-cholesterol food, salt preference, social activities, physical activity, reading, and watching TV or listening to the radio.
